# Meta-analysis of the prognostic value of long non-coding RNA AFAP1-AS1 for cancer patients in China

**DOI:** 10.18632/oncotarget.23568

**Published:** 2017-12-21

**Authors:** Rui-Hua Liu, Ming-Ying Wang, Ling-Yun Chen, Zhi-Jie Yin, Qian-Kun Ji, Yang-Yang Wang, Bao-Zhe Jin

**Affiliations:** ^1^ Department of Neurosurgery, The First Affiliated Hospital of Xinxiang Medical University, Xinxiang 453000, Henan Province, People's Republic of China; ^2^ Department of Operating Theatre, The First Affiliated Hospital of Xinxiang Medical University, Xinxiang 453000, Henan Province, People's Republic of China; ^3^ Department of Nursing, The First Affiliated Hospital of Xinxiang Medical University, Xinxiang 453000, Henan Province, People's Republic of China

**Keywords:** LncRNA, AFAP1-AS1, neoplasms, prognosis, metastasis

## Abstract

LncRNA actin filament-associated protein 1 antisense RNA 1 (AFAP1-AS1) is often dysregulated in cancer. We performed this meta-analysis to clarify the usefulness of AFAP1-AS1 as a prognostic marker in malignant tumors. The PubMed, Medline, OVID, Cochrane Library, and Web of Science databases were searched from inception to Augest 7, 2017. Sixteen studies with a total of 1,386 patients were included in the study. The pooled hazard ratio (HR) suggested high AFAP1-AS1 expression correlated with poor overall survival (OS) (HR = 1.98, 95% confidence interval (CI): 1.71–2.28), disease-free survival (DFS) (HR = 1.54, 95% CI: 1.22–1.95), and progression-free survival (PFS) (HR = 2.17, 95% CI:1.64–2.88) in cancer patients, without obvious heterogeneity. High AFAP1-AS1 expression also correlated with larger tumor size (odds ratio (OR) = 2.04, 95% CI: 1.54–2.72), advanced tumor stage (OR=2.35, 95% CI: 1.70–3.26), poor histological grade (OR =1.39, 95% CI: 1.02–1.90), lymph node metastasis (OR = 2.71, 95% CI: 1.98–3.72) and distant metastasis (OR = 2.96, 95% CI: 2.03–4.32). Thus high AFAP1-AS1 expression is predictive of poor OS, DFS, PFS, lymph node metastasis, distant metastasis, histological grade, larger tumor size and tumor stage, which suggests high AFAP1-AS1 expression may serve as a novel biomarker of poor prognosis in cancer.

## INTRODUCTION

It was recently reported that approximately 1.7 million new cancer cases and 600 thousand cancer deaths are projected to occur in the U.S. in 2017 [1]. However, the five-year survival rate of most cancers remains still low, and many scientists are looking for new biomarkers useful for diagnosis or determining prognosis in cancer.

Long noncoding RNA (lncRNA) is defined as transcribed RNA molecules greater than 200 nucleotides in length that is lack a meaningful open reading frame [2]. LncRNA has many important functions in disease, including epigenetic regulation, transcriptional and posttranscriptional regulation [3]. Moreover, it now appears that lncRNA dysregulation may be involved in various types of cancer [4–7]. For example, some lncRNAs play a key role in cancer cell proliferation, invasion and metastasis [8, 9], suggesting lncRNA may be a useful marker of cancer prognosis [10].

Actin filament-associated protein 1 antisense RNA 1 (AFAP1-AS1) is a lncRNA originally detected in esophageal adenocarcinoma [11]. It is derived from the antisense DNA strand in the AFAP1 gene locus, which regulates actin flament integrity, and acts as an adaptor protein that links Src family members and other signaling proteins associated with actin flaments [11, 12]. In addition, AFAP1-AS1 is reportedly associated with various tumor biological parameters [13–15], including overall survival (OS), lymph node metastasis and tumor stage. But although AFAP1-AS1 expression may affect prognosis and metastasis of human cancers, most studies reported so far are limited by discrete outcomes and sample size. We therefore performed this update meta-analysis to determine the prognostic value of AFAP1-AS1 in cancer patients.

## RESULTS

### Study characteristics

**Figure 1:**
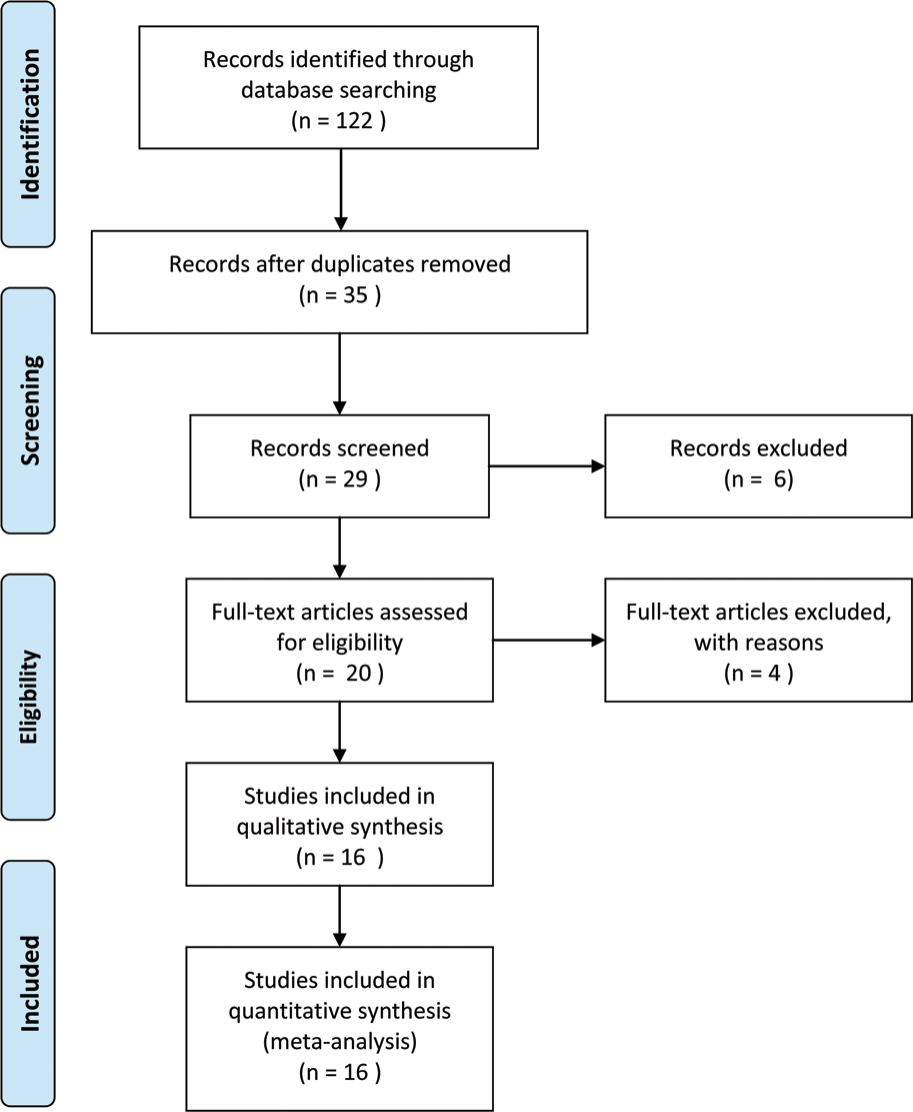
Flowchart presented the steps of study selection in this meta-analysis

The detailed screening process is shown in detail in Figure 1. According to the inclusion and exclusion criteria, sixteen studies and 1,386 patients were included in the meta-analysis [16–31]. Additionally, the characteristics of the 16 studies included in the present meta-analysis are summarized in Table 1. The subject number of 16 studies ranged from 36 to 162, with a mean sample size of 86.6. All studies were conducted in China and were published between 2015 and 2017. Among the sixteen studies, two focused on pancreatic ductal adenocarcinoma [17, 28], two focused on colorectal cancer [18, 25], two focused on hepatocellular carcinoma [19, 29], two focused on esophageal squamous cell carcinoma [21, 30], and one each on non-small cell lung cancer [16], cholangiocarcinoma [20], gallbladder cancer [22], gastric cancer [23], nasopharyngeal carcinoma [24], ovarian cancer [26], triple-negative breast cancer [27], lung adenocarcinoma [31]. All of the clinicopathological parameters were all dependent on the pathology. The reference gene of AFAP1-AS1 in these studies were found to be inconsistent, including GAPDH [17–27, 29–31], RNU48 [16] and β-actin [28].

**Table 1:** The basic information and data of all included studies in the meta-analysis

Study	Year	Region	Tumor type	Sample size	AFAP1-AS1 expression												Cut-off value	HR(95% CI) High/Low OS
					High						Low							
					Total	LTS	HTS	PHG	LNM	DM	Total	LTS	HTS	PHG	LNM	DM		
Deng [16]	2015	China	NSCLC	121	66	-	40	-	37	43	55	-	15	-	17	25	-	8.947 (3.115–25.694)
Fu [17]	2016	China	PDAC	80	40	26	20	19	18	3	40	17	18	18	16	5	Median	1.678 (0.851–3.310)
Li [18]	2016	China	CRC	30	15	-	5	-	-	-	15	-	5	-	-	-	-	3.39 (1.334–8.614)
Lu [19]	2016	China	HCC	156	78	28	65	-	-	-	78	14	51	-	-	-	Median	1.99 (1.15–3.45)
Lu [20]	2017	China	CAA	56	28	17	16	-	-	-	28	8	6	-	-	-	Median	2.31 (1.1352–4.7006)
Luo [21]	2016	China	ESCC	70	50	7	21	-	-	-	20	1	5	-	-	-	-	-
Ma [22]	2016	China	GBC	40	19	14	12	8	12	-	21	8	10	13	11	-	Median	1.95 (1.01–3.76)
Qiao [23]	2017	China	GC	87	44	33	33	23	20	19	43	30	19	16	10	8	Median	1.66 (1.1128–2.4763)
Tang [24]	2017	China	NPC	96	68	-	-	-	-	43	28	-	-	-	-	9	-	1.59 (1.2062–2.0958)
Wang [25]	2016	China	CRC	52	26	19	21	14	-	15	26	10	10	9	-	4	Median	2.358 (1.110–5.008)
Yang [26]	2016a	China	OC	130	65	33	41	39	-	-	65	27	25	35	-	-	-	-
Yang [27]	2016b	China	TNBC	102	51	3	21	-	38	8	51	1	10	-	26	1	Median	-
Ye [28]	2015	China	PDAC	90	45	-	32	13	35	-	45	-	34	9	18	-	Median	2.26 (1.48–3.44)
Zhang [29]	2016	China	HCC	78	57	27	26	39	-	-	21	6	11	8	-	-	-	1.471 (0.987–2.626)
Zhou [30]	2016	China	ESCC	162	81	28	53	23	55	22	81	25	30	22	32	9	Median	2.665 (1.838–3.865)
Zhuang [31]	2017	China	LUAD	36	20	-	-	-	-	-	16	-	-	-	-	-	-	-

Note: The dashes represent no data.

Abbreviations: NSCLC, non-small cell lung cancer; PDAC, pancreatic ductal adenocarcinoma; CRC, colorectal cancer; HCC, hepatocellular carcinoma; CAA, cholangiocarcinoma; ESCC, esophageal squamous cell carcinoma; GBC, gallbladder cancer; GC, gastric cancer; NPC, nasopharyngeal carcinoma; CRC, colorectal cancer;OC, ovarian cancer;TNBC, triple-negative breast cancer; LUAD, lung adenocarcinoma; LTS, lager tumor size; HTS, high tumor stage; PHG, poor histological grade; LNM, lymph node metastasis; DM, distant metastasis; OS, overall survival;HR, hazard ratio.

### Association between the AFAP1-AS1 expression level and survival

**Figure 2:**
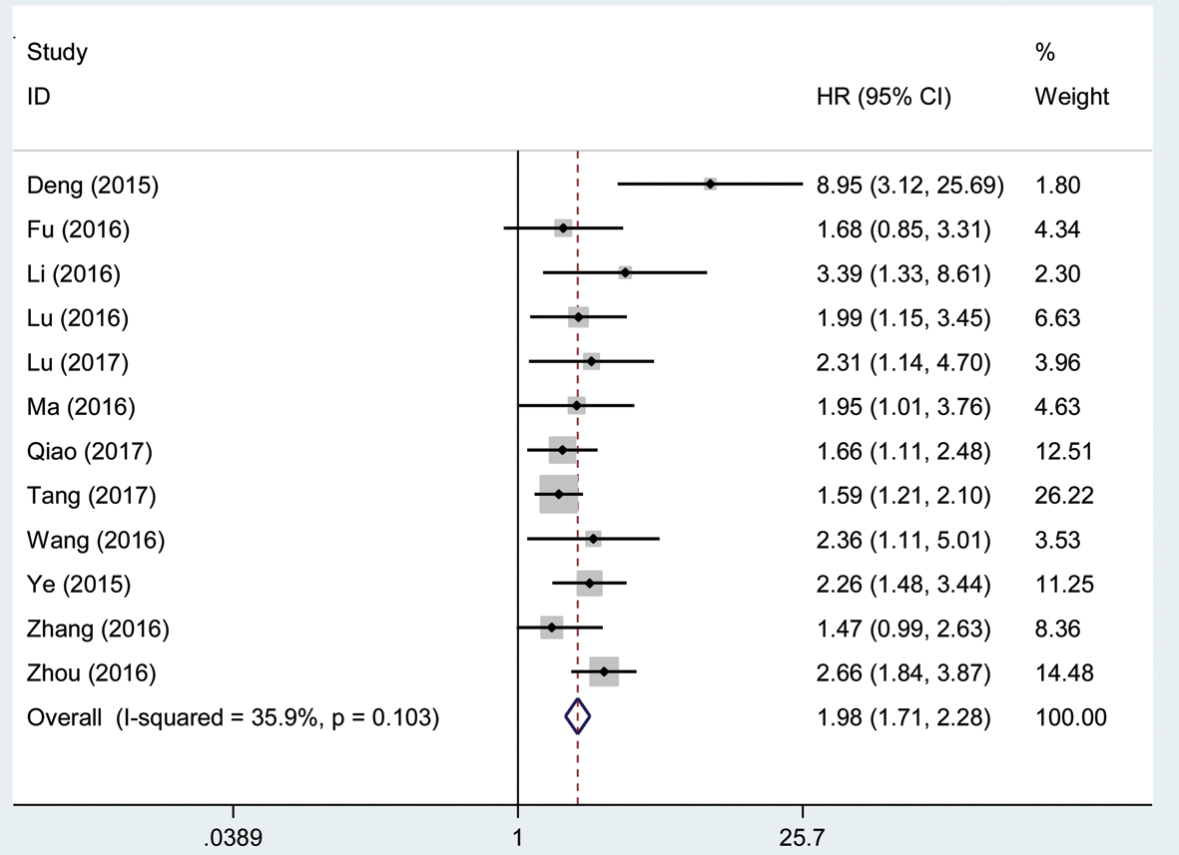
Forest plot showed the association between OS and AFAP1-AS1 expression level in cancer

We performed a cumulative meta-analysis to assess the function of AFAP1-AS1 for overall survival (OS) in patients with cancer. Additionally, twelve included studies with 1,048 patients reported the relationship between OS and AFAP1-AS1. The fix effects model was used for insignificant heterogeneity (I^2^ = 35.9%, P_Q_ = 0.103). A significant association was observed between AFAP1-AS1 and OS in cancer patients (pooled HR = 1.98, 95% CI: 1.71–2.28; Figure 2).

**Figure 3:**
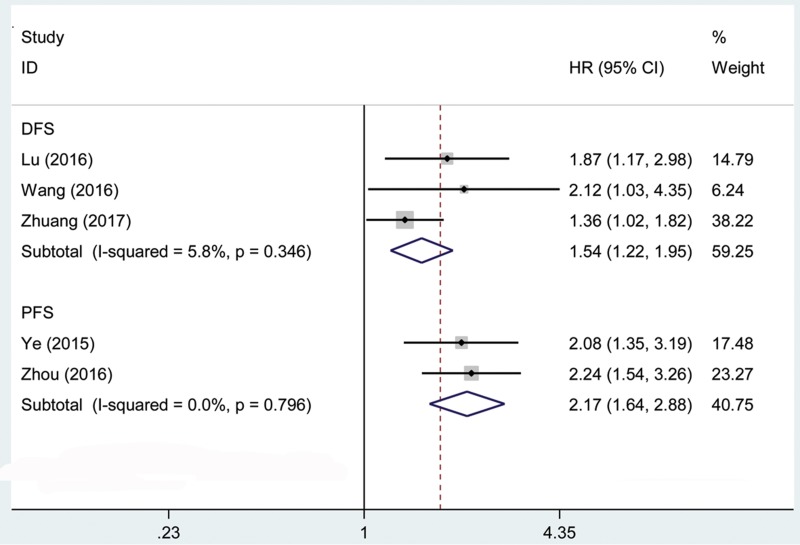
Forest plot showed the association between DFS, PFS and AFAP1-AS1 expression level in cancer

We performed cumulative meta-analysis to determine the role of AFAP1-AS1 in disease-free survival (DFS) of 244 cancer patients [19, 25, 31] and progression-free survival (PFS) of 252 cancer patients [28, 30] from the eligible studies (Figure 3). Statistical analyses revealed that AFAP1-AS1 was associated with DFS (pooled HR = 1.54, 95% CI: 1.22–1.95), PFS (pooled HR = 2.17, 95% CI: 1.64–2.88) of cancer patients. Our analyses did not find any significant heterogeneity among the studies.

This result demonstrated that a high expression of AFAP1-AS1 might be correlated with a shorter OS, DFS and PFS in cancer patients. Thus, we found that AFAP1-AS1 was an independent factor of survival among patients with cancer.

### Association between the AFAP1-AS1 expression level and tumor size

**Figure 4:**
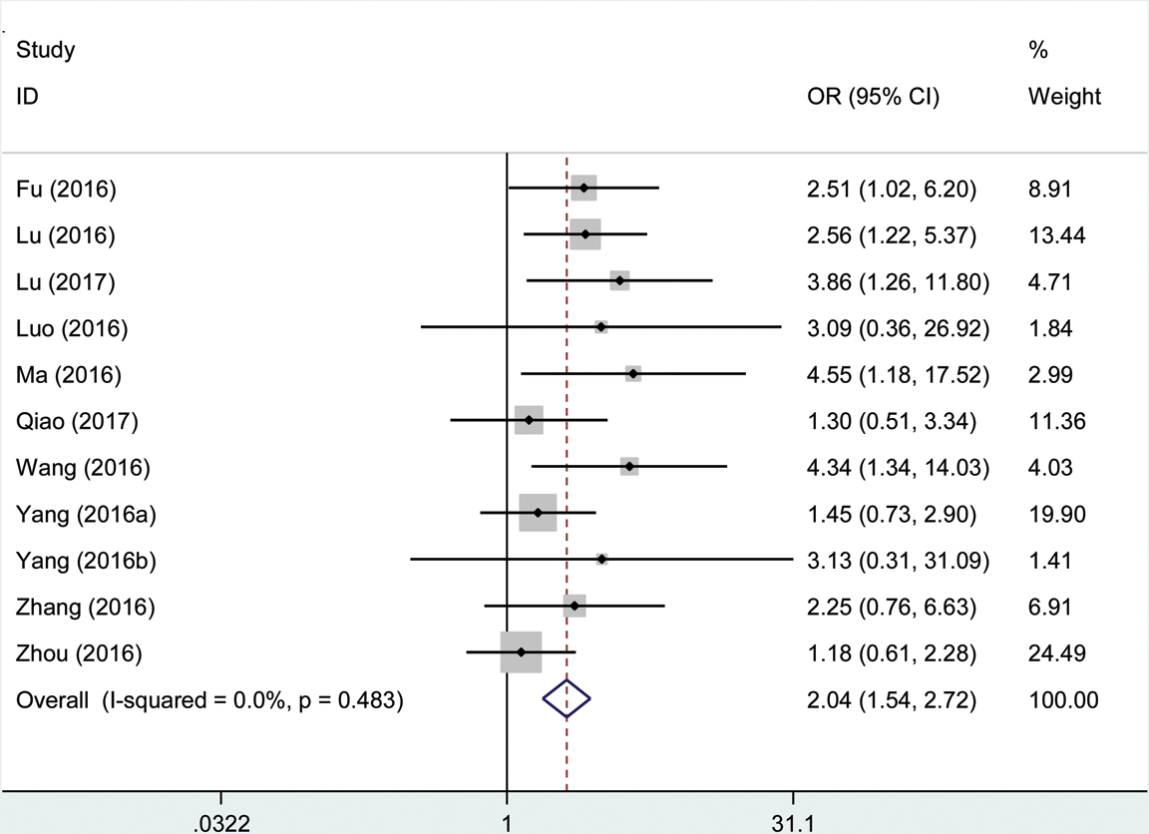
Forest plot showed the association between tumor size and AFAP1-AS1 expression level in cancer

The correlations between AFAP1-AS1 expression and tumor size are presented in Figure 4. Eleven studies with 1,013 patients declared the association between the AFAP1-AS1 expression levels and number of cancer patients with lager tumor size. There was no significant heterogeneity in these studies, and the fix-effects model was used (I^2^ = 0.00%, P_Q_ = 0.483). The analysis showed a pooled OR = 2.04 (95% CI: 1.54–2.72; high versus low AFAP1-AS1 expression; Figure 4). As a result, the patients with lager tumor size were significantly increased in the high AFAP1-AS1 expression group. The result revealed that patients with a high AFAP1-AS1 expression level in tumor tissues may indicate an increased probability of lager tumor size.

### Association between the AFAP1-AS1 expression level and tumor stage

**Figure 5:**
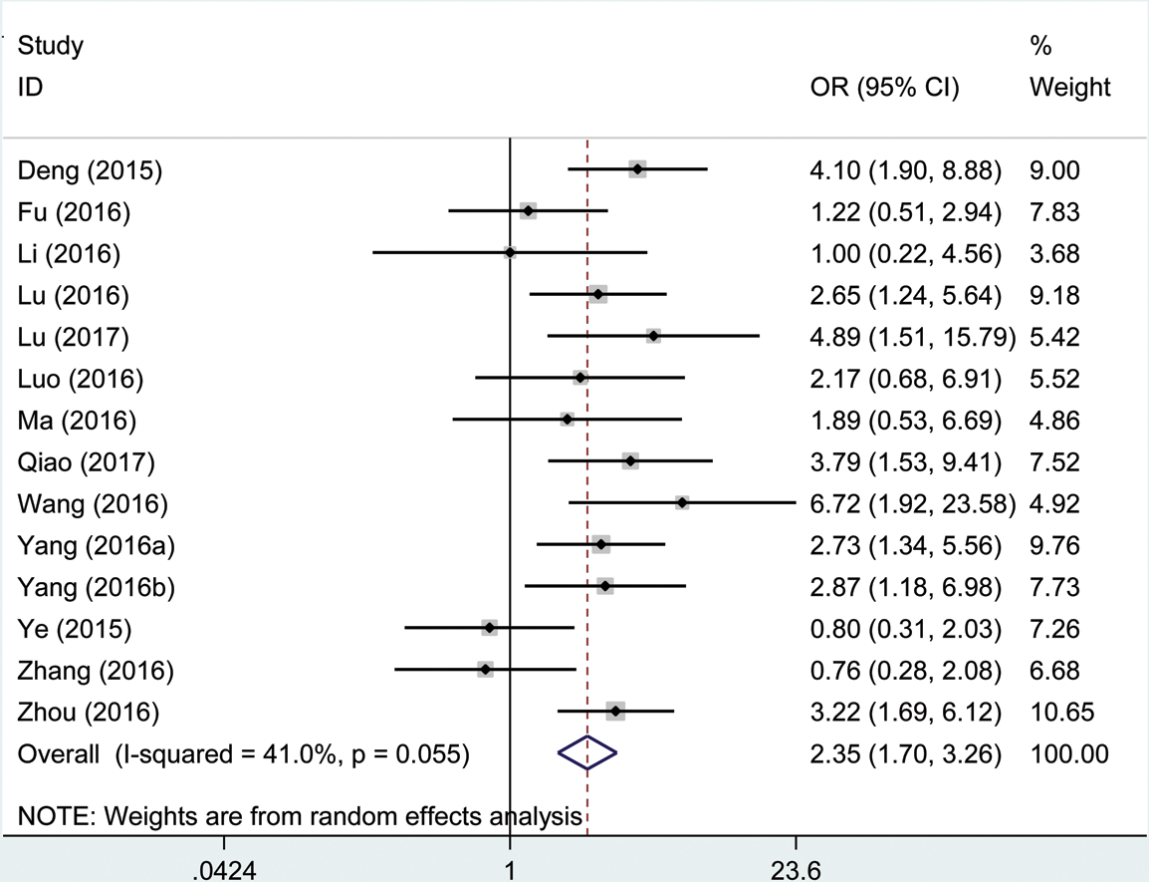
Forest plot showed the correlation between tumor stage and AFAP1-AS1 expression level in cancer

One thousand two hundred and fifty-four patients in fourteen eligible studies were included to detect the relationship between the AFAP1-AS1 expression levels and tumor stage in this meta-analysis. The random effects model was used for significant heterogeneity (I^2^ = 41.0%, P_Q_ = 0.055). A significant connection was found between a high AFAP1-AS1 expression level and high tumor stage in cancer patients (pooled OR = 2.35, 95% CI: 1.70–3.26, Figure 5). In a sensitivity analysis of all included studies, heterogeneity disappeared after the Ye, *et al.* study [28] and Zhang *et al.* study [29] were excluded (I^2^ = 0%, *P* = 0.53).

From the analysis results, the tumor stage was signifficantly increased in the high AFAP1-AS1 expression group compared with that in the low AFAP1-AS1 expression group, and the results demonstrated that a high expression of AFAP1-AS1 significantly increased the risk of high tumor stage.

### Association between the AFAP1-AS1 expression level and histological grade

**Figure 6:**
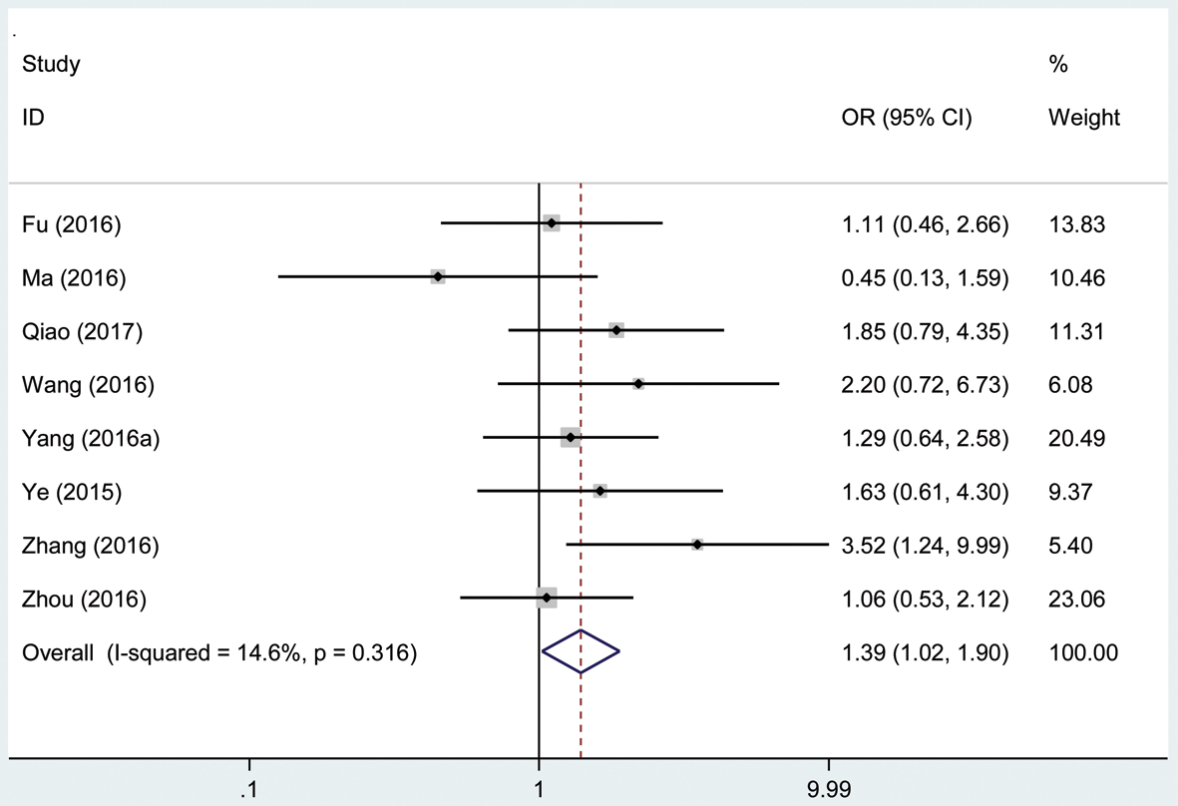
Forest plot of studies evaluated the correlation between histological grade and AFAP1-AS1 expression level in cancer

A total of 719 patients with cancer from eight eligible studies were collected and analyzed. The fix effects model was used for limited heterogeneity (I^2^ = 14.6%, P_Q_ = 0.316). The odds ratio (OR), expressed as high AFAP1-AS1 expression group versus low AFAP1-AS1 expression group, was 1.39 (95% CI: 1.02–1.90, Figure 6). According to the result, there was a significant difference in the poor differentiation grade incidence between the two groups. And the results demonstrated that high expression of AFAP1-AS1 significantly predicted more prone to poor differentiation grade for patients with cancer.

### Association between the AFAP1-AS1 expression level and lymph node metastasis

**Figure 7:**
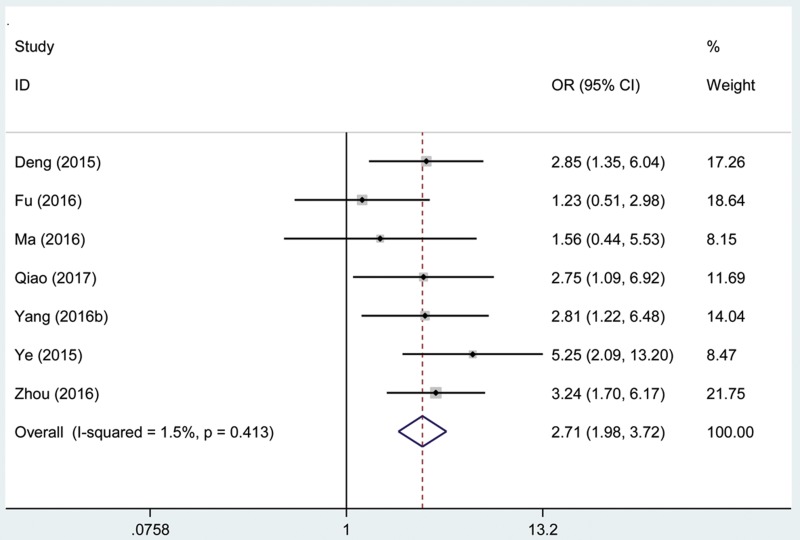
Forest plot of studies evaluated the correlation between lymph node metastasis and AFAP1-AS1 expression level in cancer

A total of 682 patients with cancer from seven eligible studies were collected and analyzed. The fix effects model was used for insignificant heterogeneity (I^2^ = 1.5%, P_Q_ = 0.413). The odds ratio (OR), expressed as high AFAP1-AS1 expression group versus low AFAP1-AS1 expression group, was 2.71 (95% CI: 1.98–3.72, Figure 7). According to the result, there was a significant difference in the lymph node metastasis incidence between the two groups. And the results demonstrated that high expression of AFAP1-AS1 significantly predicted more prone to developing lymph node metastasis for patients with cancer.

### Association between the AFAP1-AS1 expression level and distant metastasis

**Figure 8:**
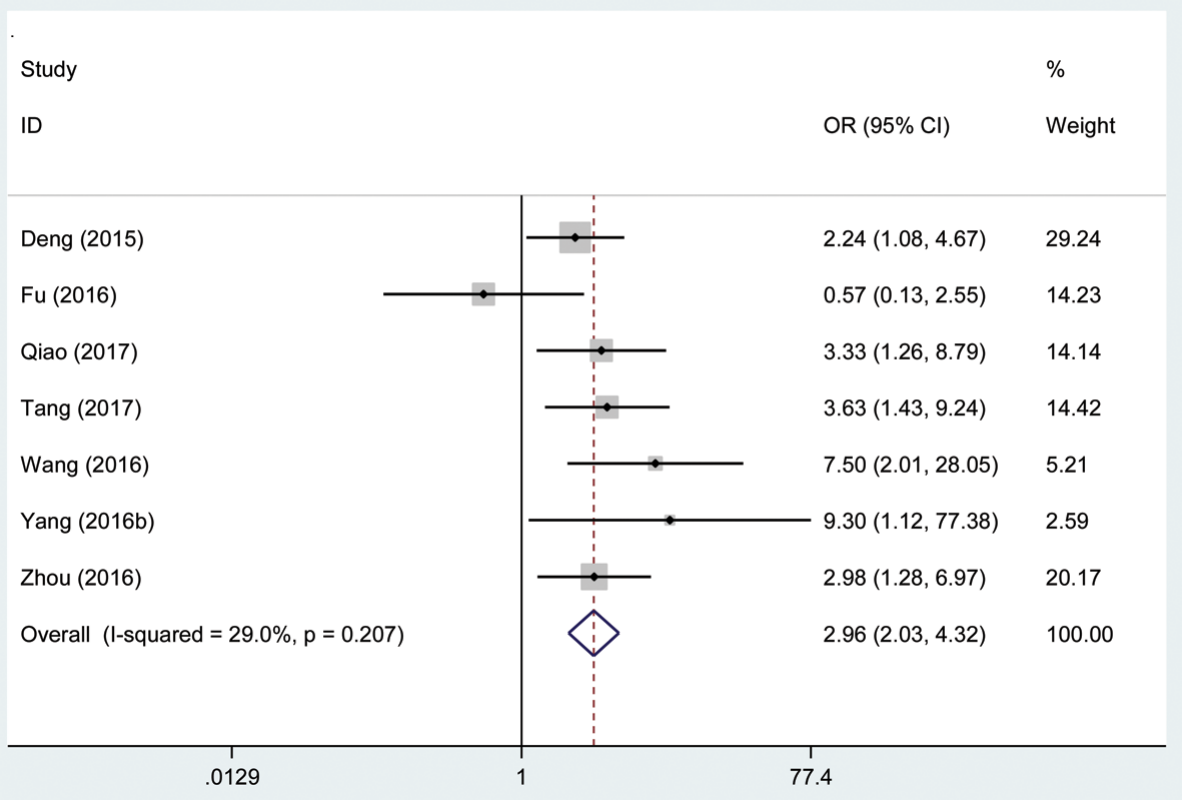
Forest plot of studies evaluated the relationship between distant metastasis and AFAP1-AS1 expression level in cancer

Seven hundred patients with cancer from 7 eligible studies were collected and analyzed. The fix effects model was used for limited heterogeneity (I^2^ = 29.0%, P_Q_ = 0.207). The odds ratio (OR), expressed as the high AFAP1-AS1 expression group versus low AFAP1-AS1 expression group was 2.96 (95% CI: 2.03–4.32, Figure 8). According to the result, there was a significant difference between the two groups in the distant metastasis incidence. Additionally, the results demonstrated that a high expression of AFAP1-AS1 significantly predicted a higher tendency to develop distant metastasis in patients with cancer.

### Publication bias

**Figure 9:**
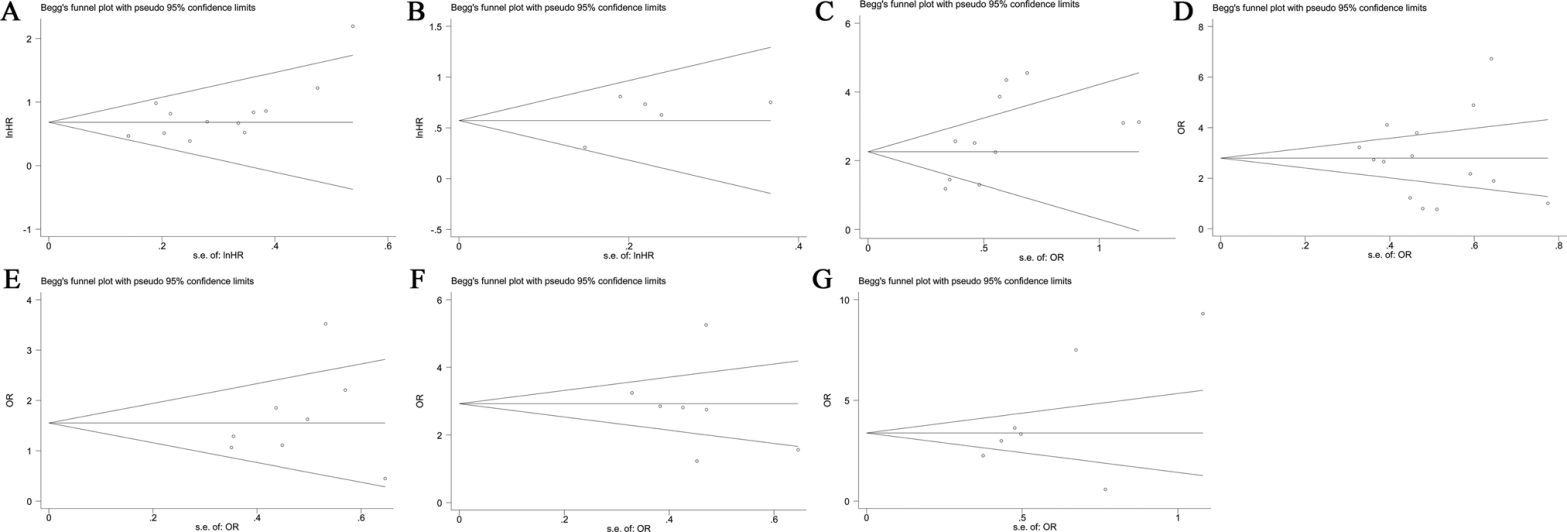
Funnel plot analysis of potential publication bias in survival and clinicopathological parameters group (**A**) OS, (**B**) DPF + PFS, (**C**) tumor size, (**D**) tumor stage, (**E**) histological grade, (**F**) lymph node metastasis, (**G**) distant metastasis.

Next, Begg's funnel plot and Egger's test were constructed to evaluate publication bias. The results showed no evidence of obvious asymmetry for OS (*P* > |t| = 0.054, Figure 9A), DPF + PFS (*P* > |t| = 0.265, Figure 9B), tumor stage (*P* > |t| = 0.872, Figure 9D), histological grade (*P* > |t| = 0.420, Figure 9E), lymph node metastasis (*P* > |t| = 0.605, Figure 9F) and distant metastasis (*P*>|t| = 0.168, Figure 9G) (Figure 9A–9BA, 9D–9G). However there was significant publication bias in terms of tumor size (*P* > |t| = 0.042, Figure 9C).

## DISCUSSION

Cancer remains a serious threat to human health, and the incidence of cancer has increased gradually in recent years [1]. The occurrence of metastasis is an important indicator of a poor prognosis [32, 33], but the precise mechanism on metastasis remains uncertain in cancer patients. At present, cancer research hotspot-molecular biomarkers play a critical role in the prediction and treatment of cancer [34, 35]. It therefore continues to be necessary to identify new molecular markers predictive of tumor metastasis. Among these molecular markers are lincRNAs, which can impact both the occurrence and development of tumors, and have shown potential to serve as easily collected biomarkers useful for diagnosing and monitoring tumors [36].

Previous studies have shown that AFAP1-AS1 is a critical oncogene in a variety human cancer types, including pancreatic ductal adenocarcinoma, cholangiocarcinoma, nasopharyngeal carcinoma, triple-negative breast cancer, lung adenocarcinoma [13–31]. Recent advances have confirmed that AFAP1-AS1 expression is up-regulated in NSCLC tissues and is associated with poor survival in NSCLC patients [16]. In addition, Luo et al [21] found that knocking down AFAP1-AS1 inhibited ESCC cell proliferation and colony-forming ability while enhancing cell apoptosis. And Yang et al [27] reported that AFAP1-AS1 is significantly overexpressed in TNBC and is associated with lymph node metastasis, distant metastasis and tumor stage.

These studies suggest that lncRNA AFAP1-AS1 may serve as an important prognostic factor in cancer patients. Until now, the underlying mechanisms by which AFAP1-AS1 affected human cancers and its utility as a biomarker remained largely unclear. Through this meta-analysis, we explored the clinicopathologic significance and prognostic value of AFAP1-AS1 in cancer patients.

A total of 1,386 patients with cancer from sixteen eligible studies were collected and analyzed in this study. A random effects model or fixed effects model was used depending on the results of heterogeneity analysis. We found that high AFAP1-AS1 expression may indicate a poor prognosis in cancer patients. By combining HRs from Cox multivariate analyses, we detected a significant difference in OS between high and low AFAP1-AS1 expression groups. We found that high AFAP1-AS1 expression was significantly associated with DFS and PFS in different types of cancer. Furthermore, high AFAP1-AS1 expression correlated significantly with larger tumor size, advanced tumor stage, poor histological grade, lymph node metastasis and distant metastasis in cancer patients. Taken together, these findings suggest AFAP1-AS1 may be a useful prognostic biomarker of poor outcome in most cancers.

### Limitations

There were several limitations that must be taken into account while interpreting the conclusions of the present meta-analysis. First, all included studies were from China. Therefore our data may not be globally applicable. Second, the included types and numbers of cancers were small. Third, seven articles did not mention the criterion used to define high expression, and the cut-off value for high expression in the remaining nine articles was the median. Therefore, additional well-designed, high-quality studies are needed to confirm the function of AFAP1-AS1 in cancer.

## MATERIALS AND METHODS

### Literature collection

According to the standard guidelines of meta-analyses [37, 38], a systematic search was performed by two authors independently in the electronic databases of Medline, Pubmed, OVID, and Web of Science for relevant articles that concerned AFAP1-AS1 as a prognostic biomarker for the survival of cancer patients. The latest search was updated on Augest 7, 2017. We performed literature search by both text word and MeSH strategy with the terms “AFAP1-AS1”, “actin filament-associated protein 1 antisense RNA 1”, “lncRNA AFAP1-AS1”, “lncRNA” or “ noncoding RNA” or “long intergenic noncoding RNA”, “carcinoma” or “neoplasm” or “tumor” or “cancer”, “prognostic” or “prognosis”, “outcome” or “survival or “recurrence”. The strategy was correspondingly adjusted in the different databases. In the retrieval process, we made a manual search using the reference lists of the relevant articles to include eligible studies.

### Study selection

Two researchers evaluated all of the included studies and extracted the data independently. The inclusion criteria were as follows: 1) the relationship between AFAP1-AS1 expression and survival was measured in multiple human tumors; 2) the expression levels of AFAP1-AS1 in human tumor tissue were measured, and the patients were grouped according to the expression levels of AFAP1-AS1; 3) all of the tumors were confirmed by pathological or histological examinations; 4) studies statistically analyzed patient survival or pathological parameters such as lymph node metastasis, tumor size and tumor stage, with respect to AFAP1-AS1 expression.

The following studies were excluded: 1) reviews, letters, editorials, and expert opinions; 2) non-English language and non-human studies; 3) studies with the molecular structure and functions of AFAP1-AS1 only; 4) database analysis without original data.

### Data extraction

Two reviewers extracted and examined the data from the original articles independently. Disagreements in the literature assessment were resolved through consensus with a third reviewer. The following data were collected: surname of the first author, publication year, country, tumor type, sample size, the number of patients with lager tumor size, poor histological (differentiation) grade, high tumor stage, lymph node metastasis and distant metastasis, description of the cut-off value of AFAP1-AS1, HR and 95% CI of elevated AFAP1-AS1 for OS.

### Statistical methods

Statistical analyses were performed using Stata version 12.0 software. The heterogeneity among different studies was measured by the Q and I^2^ tests. A probability value of I^2^ ≥ 50%, and *P* < 0.1 indicated the existence of significant heterogeneity [39]. A random effects model or fixed effects model was used depending on the results of heterogeneity analysis. If there was a significant heterogeneity among the studies, the random-effects model was adopted. The potential publication bias was assessed by the Begg's funnel plot and Egger's test. Pooled HRs and ORs were extracted from the published data. If the HRs can be obtained directly from the publication, we used crude ones. While the HR and 95% CI were not directly reported in the studies, survival information was extracted from Kaplan-Meier curves and was used to estimate the HR. The log HR and SE were used to summarize the outcome of survival [40]. OR and their 95% CI were combined to assess the association between AFAP1-AS1 expression and clinicopathological parameters, including tumor size, histological (differentiation) grade, tumor stage, lymph node metastasis and distant metastasis.

## CONCLUSIONS

In sum, high levels of AFAP1-AS1 expression in multiple cancers is significantly correlated with poor OS, DFS, PFS, LYMPH NODE METASTASIS, DISTANT METASTASIS, histological grade, larger tumor size, and tumor stage. Therefore, AFAP1-AS1 expression may serve as a promising biomarker for predicting prognosis and metastasis in cancer patients.
